# Channel branching and zigzagging in negative cloud-to-ground lightning

**DOI:** 10.1038/s41598-017-03686-w

**Published:** 2017-06-14

**Authors:** Rubin Jiang, Xiushu Qie, Hongbo Zhang, Mingyuan Liu, Zhuling Sun, Gaopeng Lu, Zhichao Wang, Yu Wang

**Affiliations:** 1 Key Laboratory of Middle Atmosphere and Global Environment Observation (LAGEO), Institute of Atmospheric Physics, Chinese Academy of Sciences, Beijing, 100029 China; 2 Collaborative Innovation Center on Forecast and Evaluation of Meteorological Disasters, Nanjing University of information Science & Technology, Nanjing, 210044 China; 3 University of Chinese Academy of Sciences, Beijing, 100049 China; 4 Wuhan NARI Limited Company, State Grid Electric Power Research Institute, Wuhan, 430074 China

## Abstract

A fundamental question in lightning flash concerns why the discharge channel propagates in a zig-zag manner and produces extensive branches. Here we report the optical observation of two negative cloud-to-ground lightning discharges with very high temporal resolution of 180,000 frames per second, which shows in detail the dependence of channel branching and tortuous behavior on the stepping process of the leader development. It is found that the clustered space leaders formed in parallel ahead of the channel tip during an individual step process. The leader branching is due to the multiple connection of the clustered space leaders with the same root channel tip, which occur almost simultaneously, or successively as some space leaders/stems resurrect after interruption. Meanwhile, the irregularity of angles between the clustered space leaders and the advancing direction of leader tip is the origin of channel tortuosity. The statistical analysis on 96 steps shows a geometric-mean value of 4.4 m for the step length, ranging between 1.3 and 8.6 m, while the distance from the center of space leader to the channel is 3.6 m, ranging between 2.1 and 6.9 m. More than 50% steps occurred within an angle range of ±30° from the advancing direction of the leader.

## Introduction

The long brilliant discharge channel of lightning flash in atmosphere spreads several to tens of kilometers with extensive branches, making it a majestic scene during thunderstorm event. In the study of lightning physics, the fundamental issue of how lightning propagates and branches has raised wide and long-standing concerns from researchers around the world^1–5^. However, although lots of optical images on lightning flashes were captured and their stationary fractal characteristics were analyzed carefully^6–8^, the physical mechanism for the formation of channel branching and zigzagging in lightning still remains unclear. In general, one may attribute this random feature to the non-uniformity and irregularity of the virgin air through which the lightning breaks down, but it is far from clarity and adequacy to interpret such fractal phenomenon in atmosphere, especially considering that the air properties along and in front of the discharge channel always fluctuates during the extension of leader channel as the lightning develops.

In negative cloud-to-ground lightning discharges, the fact that the negative leader breaking down virgin air propagates in a stepwise manner is well known, as photographed by streak camera in early days^9, 10^, and by high speed video cameras more recently^5, 11–14^. Schonland^15^ pointed out that the tortuous path of negative lightning channel was due to the quasi-arbitrary pointing of successive steps, and the branching occurs with the division of a step into a fork. To date, however, although the physical mechanism of stepped process in negative leader has been well investigated, mainly from the laboratory long sparks and partly from the observations of triggered and natural lightning, the dynamic process that concretely leads to branching and its dependence on the specifics of step development are still not very clear. Some recent findings are found in Tran *et al*.^14^ showing concurrently extending branches as the stepped leader of an unusual lightning flash propagated. In this paper, we further report on optical recording of two negative stepped leaders with very high temporal resolution of 180,000 frames per second (fps), which promotes detailed analysis on the leader branching and zigzagging behavior.

In order to make the analysis and results more logical and understandable, with some mentioned parameters or terms comparable to those from references, here we give a very brief review on the sketchy mechanism for the development of negative stepped leader, as mainly revealed by long sparks observations in laboratory, with the discharge environment adjustable^16–18^. Within a step cycle, corona streamers in negative polarity firstly initiate at the channel tip of the leader, heating the traces. Afterwards a pilot system generates, of which the coronas in positive polarity propagate backward to the tip of the leader while the coronas in negative polarity propagate forward, both coronas initiate from a common root known as the space-stem. Such space-stem progressively grow and heat, evolve as space leader with detectable luminosity and extend bi-directionally. Eventually, the bidirectional space leader realizes the junction to the existing channel tip and accomplishes a stepping cycle, producing impulsive current accompanied with channel jump of both luminosity and length.

Since the long sparks occur in different discharge environment from the natural lightning, their charge source and discharge length scale are not the same, great uncertainty exists when employing the findings from laboratory to interpret the properties of natural lightning. More recently, a few high-speed optical observations have added observational facts on some important aspects, such as the luminous segments of meters-scale ahead of the leader tip, representative of the space leaders^11–13, 19, 20^. By identifying two adjacent space stems ahead of the leader in a high-speed image at 2500 fps, Tran *et al*.^14^ considered that they may give rise to the branched channel extension. As further indicated by our observation, clustered space leaders formed in parallel in front of the leader tip is a common phenomenon during a step cycle, their successive connection to the tip leads to the splitting of leader into a fork.

## Results

**Figure 1:**
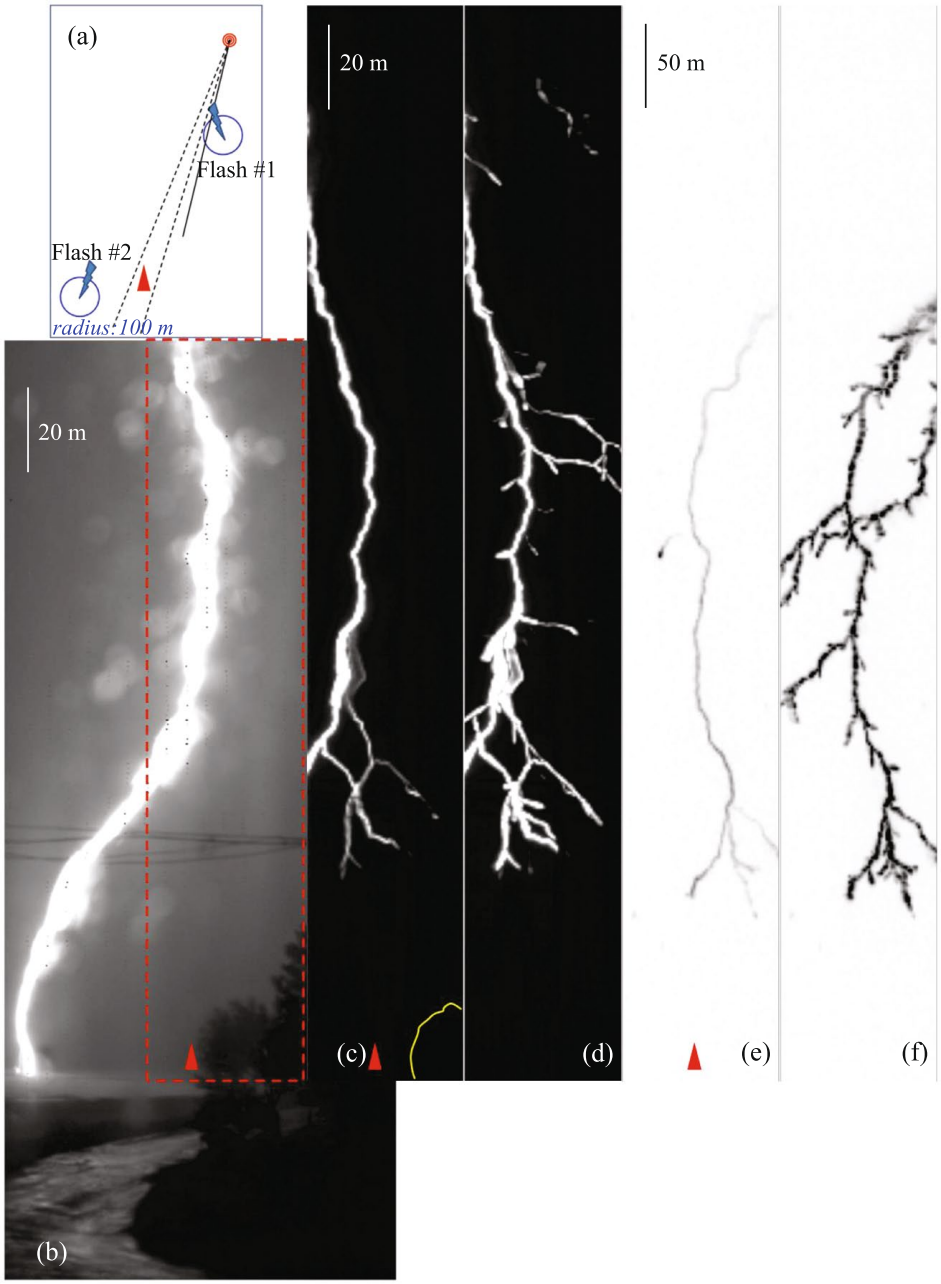
(**a**) Sketch of the observation site with the flash locations marked (**b**) a frame of Flash #1 obtained by the M310 camera at 11,000 fps (**c**) the frame just prior to the return stroke of Flash #1 obtained by the V711 camera at 180,000 fps (**d**) the time-integrated image for the stepped leader development of Flash 1# as it propagated within the field of vision of the V711 camera (**e**) same as image-c but for Flash #2; and (**f**) same as image-d but for Flash #2. The red dotted box overlaps the field of view of the V711 camera with that of the M 310 camera, and due to a 3-m horizontal distance between the cameras, there is a very tiny difference in their field angles. The black dashed lines indicate the field angle of the V711 camera.

Two natural cloud-to-ground lightning flashes occurring at 17:23:07 UTC on 11 August (Flash #1) and 11:59:56 UTC on 12 August (Flash #2), 2013, were partly captured by our high-speed digital video cameras, with horizontal distances of 410 m and 1030 m, respectively, as shown in Fig. 1. Both flashes are of negative polarity based on the associated electric field change measurement. The images with temporal resolution of about 5.6 µs (at frame rates of 180,000 fps) are used in the following analysis.

### Clustered space leaders and initiation of leader branching

**Figure 2:**
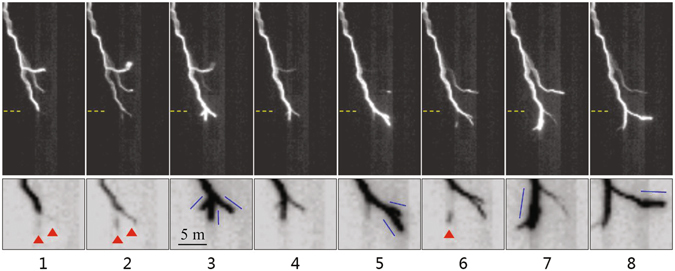
Eight consecutive frames for Flash #1 that reveal the stepped leader evolution. The frames have a 5.56-μs temporal resolution (obtained at 180,000 fps with exposure time of 5.18 μs).

Figure 2 shows eight consecutive frames (clipped) of Flash #1 for illustrating the evolution of leader stepping, including the appearance of space leader, the abrupt jump of the channel, the following pause, and then the next step development. The bottom panel shows an expanded view of the tip area of the leader channel in inverted color. In frames 1, 2, and 6, luminous segments apart from the leader tip are captured, indicative of space leaders, as marked with red triangles. Here we consider the segments as space leaders rather than space stems because of their measurable luminosity, which indicates that they have already been heated by the corona currents.

The fact worth emphasizing is that two segments ahead of the same channel tip are simultaneously found in frame 1, although they are relatively weak in luminosity at that time. The distances from the centers of these luminous segments to the tip are almost the same (the right one seems slightly closer to the tip). In frame 2, both segments grow in length and exhibit brightened luminosity, indicative of a development and enhancement of space leaders. Then in frame 3, as the stepping process is accomplished, the channel splits into three branches with new tips pointing toward different directions. It means that three separate space leaders have made junction to the original channel tip very close in time, although the space leader on the left is not captured in these sequential images. It is possible that the development duration of this space leader (with measurable luminosity after being strengthened from a space stem) is shorter than 5.6 µs, the time resolution of the images, or, its luminosity is too weak to be detected. Based on the current frame rate, the possible propagation of the space leaders (the backward portion) toward the leader tip is not evident, but is inferred from the previously known phenomenology as mentioned above, and the temporal order of space leaders’ attachment to the channel is not recognized. Nevertheless, it is definitely sure that the attachments occur within a same individual step development, and the time difference should be quite short.

In frame 4, the leader channel appears paused, with a reduction of channel luminosity, especially at the newly formed channel tips. The channel does not extend any longer in this frame, and no luminous segment is imaged. Then in frame 5, a new leader-step develops, at the front of the right branch that is just generated. It seems that the space leader development for this step is too rapid for the camera to resolve at the current frame rate. The result of this step is that the just generated right branch further split into two branches. Based on the successive frames 4 and 5, and taking the leader stepping mechanism into account, it is reasonable that the un-imaged space leaders had occurred in parallel and then made attachment with the right branch that was just generated in frame 4.

**Figure 3:**
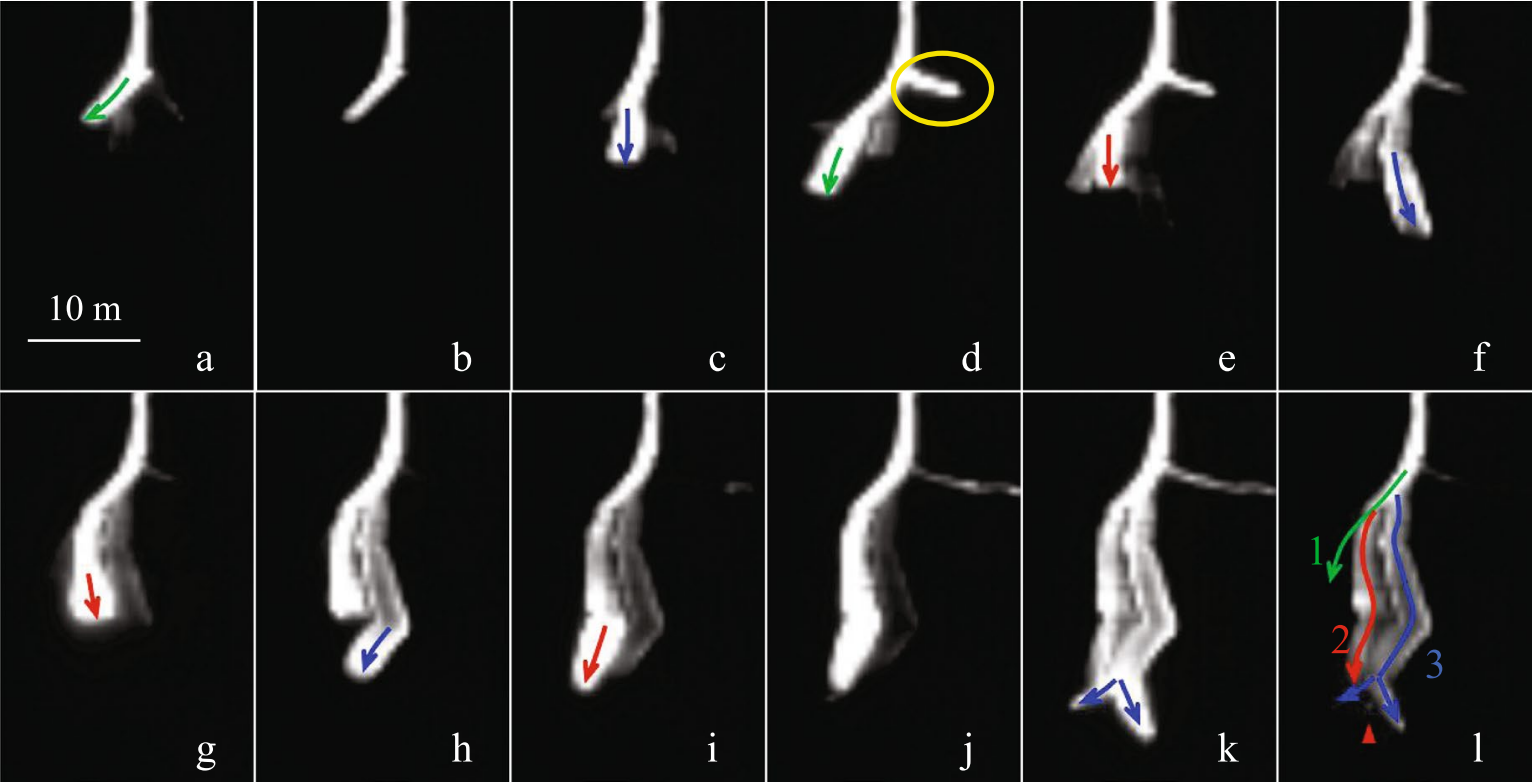
Consecutive frames for Flash #1 illustrating the alternate stepping of the branches sharing a root. The green, red and blue arrowed curves indicate the stepped development of branches 1, 2 and 3, respectively.

Frames a-d in Fig. 3 further shows the leader branching and the stepping behaviors. In frame-a, a step process is accomplished due to the connection of the space leader on the left side to the pre-existing channel. This frame also captures the appearance of the space leaders to the middle and the right side, which has not yet realized the connection and shows much weaker luminosity. In frame-b, these two unconnected space leaders seem to be extinguished, or, interrupted, as they disappear in the image. In the following frame-c, a new step occurs in the area of the previous middle space leader, indicating a re-activation of such interrupted space leader and its following connection to the existing channel, although the junction point is not the newly generated channel tip as shown in frame-b. Then in frame-d on the right side, a new step (marked with yellow circle) occurs in an area where no previous luminous segment has ever been captured. Nevertheless, the junction node connected by this step is the same with the node shown in frame-a, with a stepped channel extension already on the left and a weak space leader (extinguished later) on the right side. Since the junction node is identical, it is rational that in frame-a, three (or even more) space stems/leaders have once been developed, with some un-imaged, for example the one in the yellow circle that leads to the newly formed step. There is a lagging time of nearly 20 µs after the un-imaged space stem has been interrupted.

**Figure 4:**
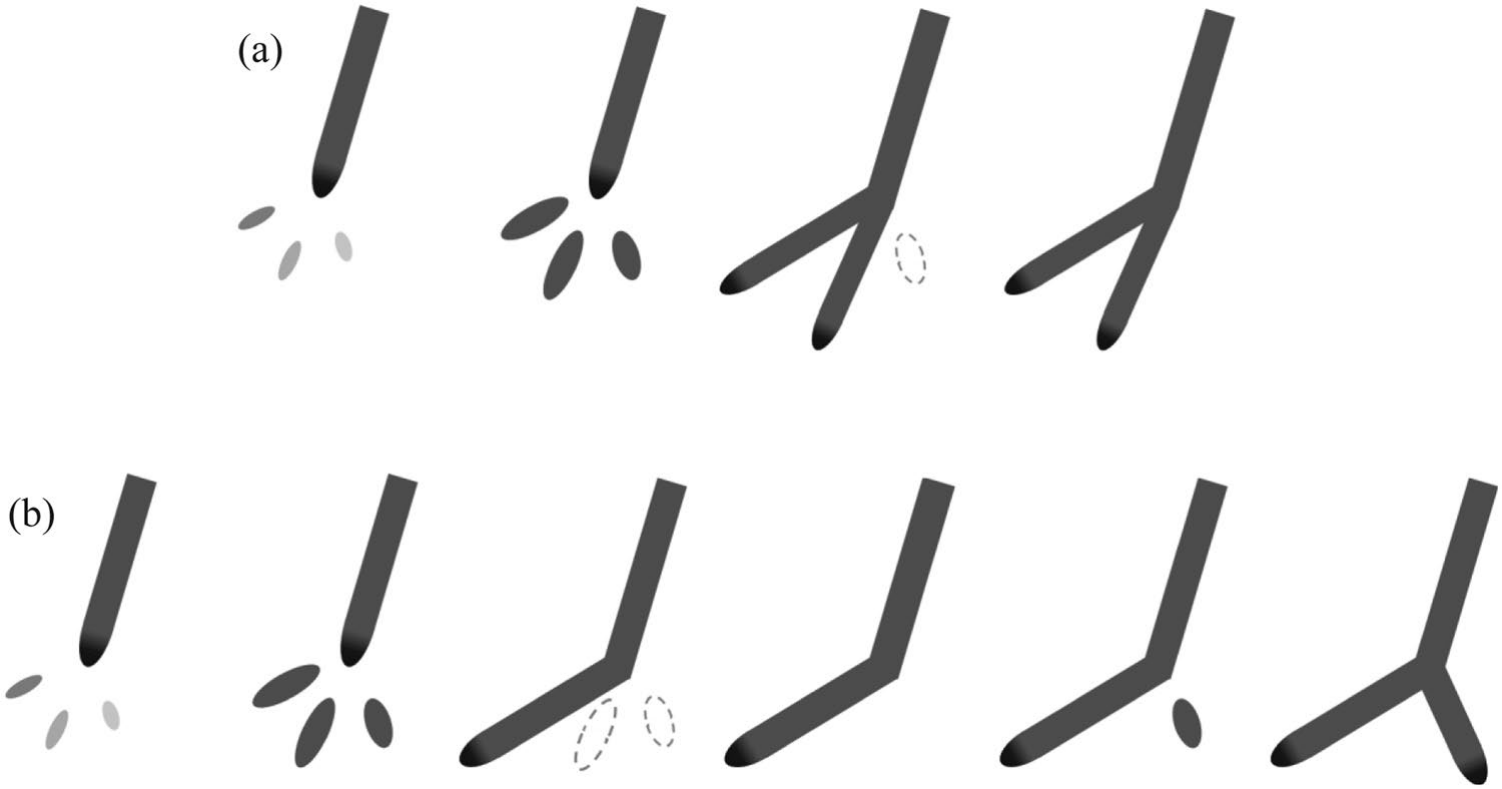
Sketch diagram of two kinds of leader branching mechanism, (**a**) occurrence of multiple space leaders and their connection to the same channel tip within a step cycle, (**b**) multiple space leaders and the re-activation of an interrupted space leader after a step towards a different direction has previously occurred.

The above analysis confirm that there is a great dependence of the negative leader branching on the stepping development, and reveal that two scenarios can both eventually result in leader branching: the first is that the multiple space leaders connect to a same channel tip very closely in time within a same individual step process, the second is that the re-activation of an interrupted space leader/space stem connects to the node where a step towards a different direction has previously occurred. These two kinds of leader branching mechanism are schematically shown in Fig. 4. It deserves emphasizing that both these processes are based on the prerequisite that multiple space stems/leaders formed in parallel in front of an individual channel tip during a step cycle. The last two stages of the Fig. 4 were once discussed in a recent study by Qi *et al*.^13^ when making sketches of leader step formation process. This further indicate that the branching is resulted from the stepping.

### Alternate stepping of sub-branches from a common root

As the leader channel splits into two or three branches in virtue of the stepping development, the newly formed sub-branches will further extend in stepwise manner. It is interestingly found that the emerging tips of a freshly branched negative leader appear to step alternately (or sequentially) rather than simultaneously, during their initial extension from the branching node. For the three branches formed in the frame 3 of Fig. 2, the left one is eventually extinguished while those in the middle and right continue to progress. In frame 4, the whole leader channel pauses and the sub-branches exhibits dimmed luminosity, then the right sub-branch steps again by the junction of two newly space leaders to the associated tip (frame 5), while the branch in the middle remains at a standstill. Sequentially in the following two frames, the right sub-branch pauses while sub-branch in the middle accomplishes a new step development, with the emergence of a space leader at front and then the occurrence of the connection. Then the following frame 8 shows that the right sub-branch steps one more time. During such alternate stepping, the new step results in a significant enhancement of luminosity in the associated branch, while contrarily, the pausing branch emits weakening luminosity.

Figure 3 also illustrates the sequential stepwise extension of freshly generated tips of the negative leader after branching. For branches 1, 2 and 3 (refer to frame l), during their initial extension in these 12 consecutive frames, the order of their stepping is: 1 → 3 → 1 → 2 → 3 → 2 → 3 → 2 → 3, i.e. alternating developing. At the current time resolution of video frames, it is generally found that such alternate behavior won’t become less pronounced until the branches develop into sufficient lengths or divide into more sub branches.

### Characteristics of leader steps and channel tortuosity

**Figure 5:**
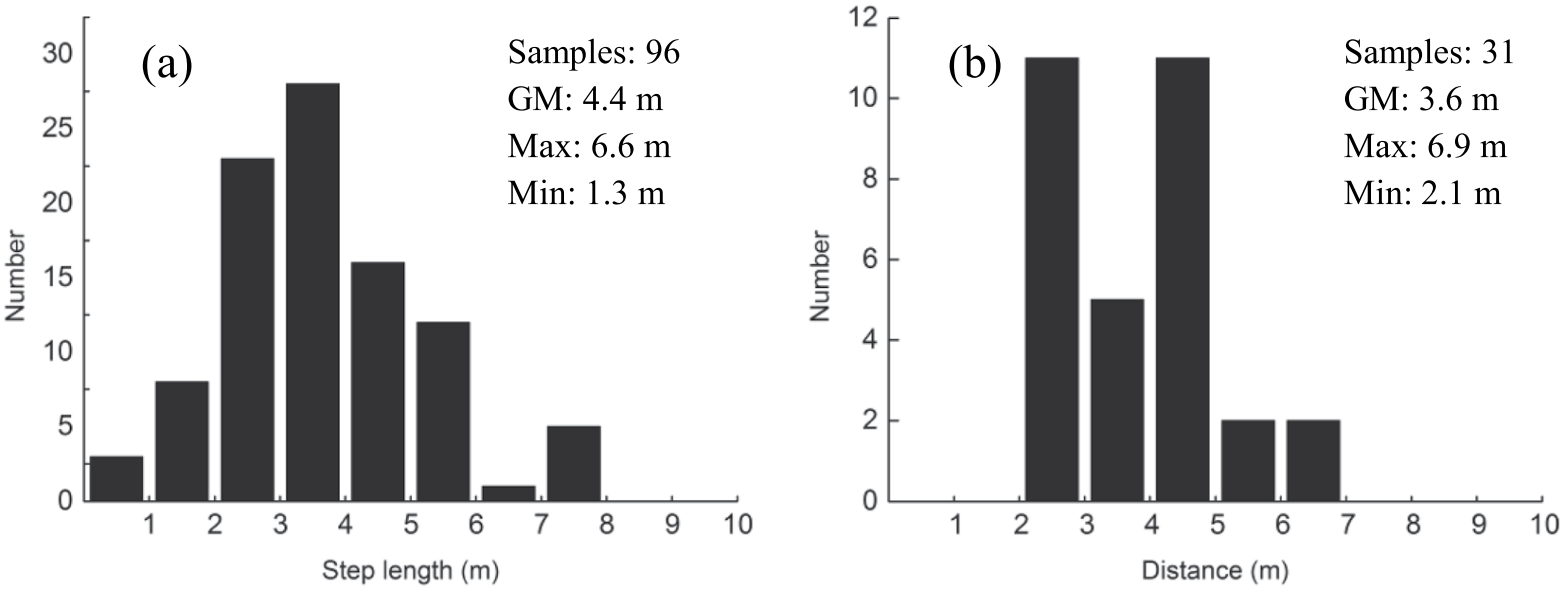
Histograms of (**a**) step length, and (**b**) distance from the center of space leader to the channel tip.

During a period of about 667 µs with 120 sequential frames, a total of 96 individual stepped-channel-extensions are recognized for Flash #1 with a relatively close distance from the camera, and meanwhile, 31 space leader segments are identified, four of which maintains luminous in two successive frames. The 2-D length of individual step and distance from the space leader center to the channel tip are calculated. Figure 5 shows the distributions of these two parameters. The step length ranges from 1.3 m to 8.6 m and the geometric mean is 4.4 m (4.6 m on average). For 31 samples of the distance between the center of space leader and the channel tip, the geometric mean is 3.6 m (3.8 m on average), and it varies in the range of 2.1–6.9 m. Since the space leader with detectable luminosity lasts a very short time while evolving from space stem and it persistently grows in length, it does not make much sense to analyze the segment length or the gap length between the segment and the main channel at the moment when the segment is captured in a frame. These two values may vary as the space leader develops. By comparing the 2-D results in this paper with the previous observations^5, 12–14, 21, 22^, the step-length herein is more or less consistent with that obtained by Hill *et al*.^11^, giving an average value of 5.2 m under an estimated observing distance of 1 km. Biagi *et al*.^21^ reported five steps in an altitude-triggered lightning to range in 5–8 m, with similar upper value to our result. By using a photodiode array photographic system, Chen *et al*.^22^ observed two negative leaders with mean step length of 13.3 m (ranged in 7.9–19.8 m) and 8.5 m, respectively, larger than the results in this paper.

**Figure 6:**
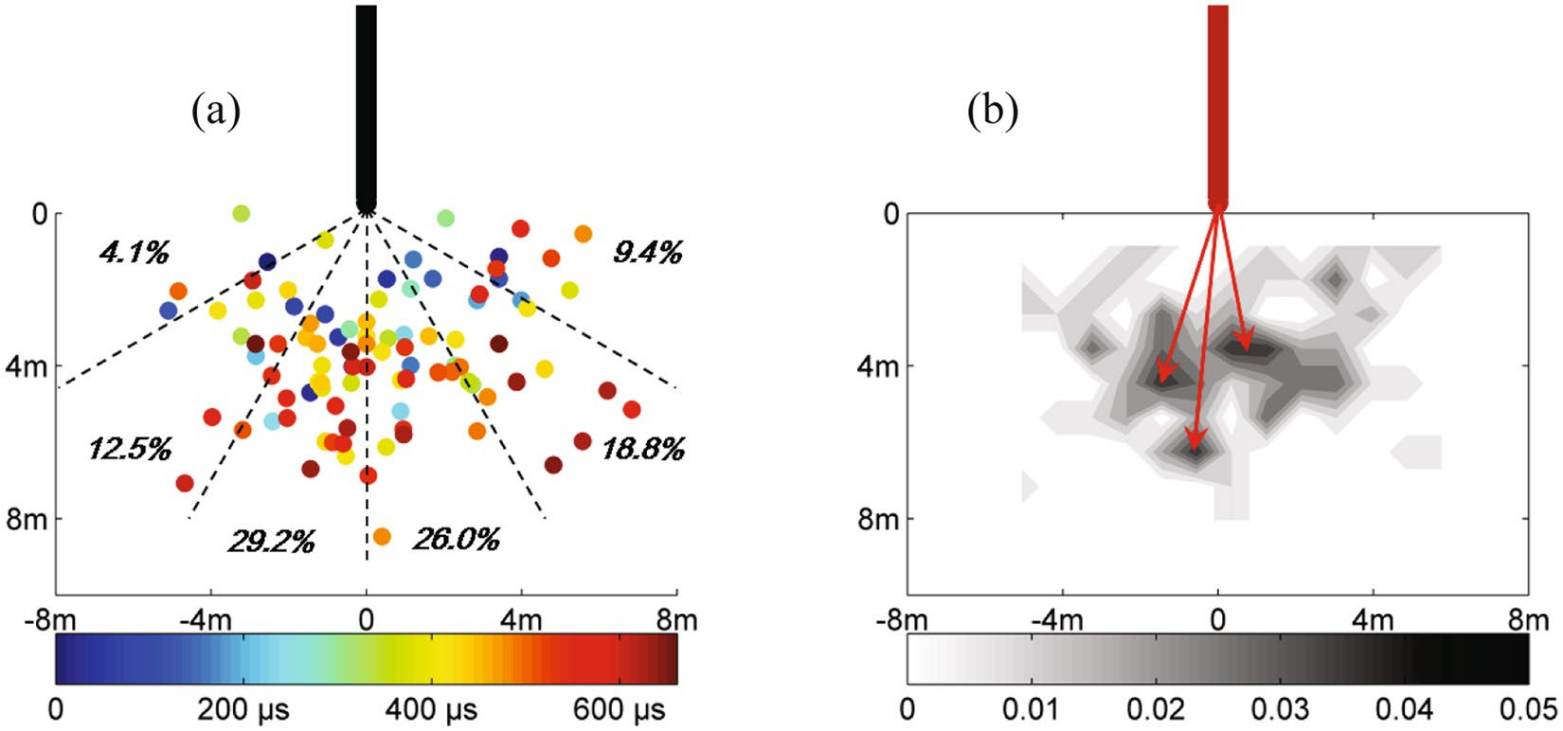
(**a**) Scatter plot showing the locations of each individual steps with the distance and relative direction from the channel tip, (**b**) probability density distribution of the steps.

Figure 6a further shows the scatter plot of the 96 steps relative to the tip of the leader trunk. The scatter points represent the direction and step length (distance from the tip) of each individual steps as they jumps downward from the descending channel tip, and the color bar gives the relative time of their occurrence during a 667-µs period. The direction (or the angle) of the scatter points relative to the leader tip shown in the figure is obtained by comparing the new step with the prior step, which means a relative angle in regard to the advancing direction of the existing channel when the new step occurs by junction of space leader to the tip. As indicated in the figure, the direction of the steps is quite random, with the maximum relative angle up to about 90°, the straight forward direction from the tip does not involve significant ascendency for an individual step to jump towards. Nevertheless, within an angle range of ±30° ahead from the channel tip, the step probabilities are indeed larger. More than 50% of all the steps occurs in such a 1/3 region in front of the channel tip. It seems that the step-length of the leader does not exhibit an obvious increasing tendency as it gets closer to ground with time, although in the figure, the blue points are a little more concentrated to the channel tip as compared to the red points. Quite a few steps just before the occurrence of return stroke involves relatively larger step length.

Based on the scatter point positions, the probability density is calculated, with the distribution shown in Fig. 6b. The grayscale indicates the probability of a step-jumping from the tip (0 m, 0 m) to the grid coordinates. There are three peak density areas in the figure, marked with the red arrows, indicating that the most likely step-length and jump-direction for a step in this leader development are 3.9 m at −10.8° angle, 6.5 m at 5.7° angle, or 4.9 m at 17.9°. Note that the clockwise angle is assigned as positive.

The randomly dispersed feature of the scatter plots in Fig. 6a indicates that during every tiny stepped channel extension of the leader, a variation in direction will be superimposed on the overall downward propagation to ground, which is determined by the overall potential difference between the charged cloud and the ground. This makes the leader channel a tortuous pattern. That is, the leader propagates in zig-zag way because very individual step development of the leader exhibits randomly changing direction.

## Discussion

By integrating luminosity of the sequential images during leader developments, abundant tiny branches through the leader channels are revealed, as shown in Fig. 1d (Flash #1) and 1f (Flash #2). This indicates that the leaders have branched for a lot of times during their downward propagation, much more than what we observed in a certain frame that shows just a few branches, such as the frames shown in Fig. 1c,e. Numerous tiny branches are produced during the stepping development of the leader and most of them do not propagate far from the main channel. They extinguish shortly after generation, leading to lots of nodes with short luminous segments along the channel path in the integrated image. Since the leader-branching is due to the clustered space leaders (which are strengthened from some of those multiple space stems) connecting to the same channel tip very close in time or successively, and meanwhile, the numerous branched nodes along the leader channel certify that the branching occurs frequently, it is rationally valid that the multiple space leaders are a common phenomenon during the step development of the natural lightning leader, although they could not be easily captured in sequential images by using cameras with relatively slow frame-rate. Actually, Tran *et al*.^21^ once captured two concurrent faint luminous formations ahead of the main channel tip with a frame rate of 2500 fps, but the images did not sufficiently show the branch formation in detail.

When discussing the development of space leaders with short time duration which are not easily captured, an important fact also needs to be objectively noticed. The developments of stems and even streamers are cold and rather dark discharges in the visible range, especially considering a distance of several hundreds of meters from the leader to the camera. But their occurrences determine the behavior of space leaders, and eventually determine the stepping process of the leader. Being unable to reveal the streamers is a limitation of optical data in investigating the leader properties. Nevertheless, since the space leaders are evolved from the space stems, by confirming the common occurrence of multiple space leaders, some new insight can still be obtained, such as the tortuous path of lightning leader channel. When generating in front of the channel tip, those clustered space leaders would extend bi-directionally, and there would be a competitive relationship for them to make connection with the existing channel. As shown in frames a and b of Fig. 3, the firstly occurring step in the left, has lead to the abortion of the right space leaders and the interruption of the middle one. Similar phenomena are also observed during other step processes. As illustrated in the third phase of Fig. 4a,b, the old leader tip becomes a part of the channel after the accomplishment of a step, which means it does not “sharp” anymore. The transient increase of the equivalent radius of curvature results in a sudden decrease of the electric field surrounding the old leader tip region. Correspondingly, those unconnected space leaders would lose the supporting background energy and become aborted or interrupted. Most of the aborted space leaders are entirely extinguished as the leader propagates further downward, while some of the interrupted ones re-start during the following step process. Since the clustered space leaders in parallel involve different relative angles to the channel tip, the quasi-random direction of the successive steps lies in the uncertainty for one or two of the clustered space leaders to enhance dominantly. Such uncertainty and randomness eventually make the lightning a fractal pattern.

**Figure 7:**
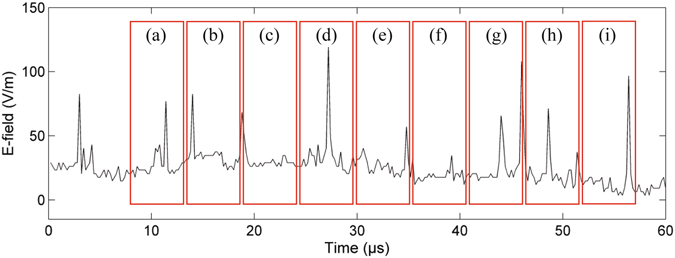
The surface electric field changes of the leader development shown in Fig. 3, with exposure time of each frames marked with red boxes.

As an electrical discharge process, the leader properties will be reflected in the electric field measurement. Figure 7 shows the surface electric field change due to the leader development of Flash 1# as shown in Fig. 3. The red boxes mark the exposure time when the frames were taken, on bases of the GPS synchronization. As shown in the figure, the impulsive changes associated with the leader steps are evident, which is due to the connection of the space leaders with the existing channel. There are two facts that are worth emphasizing. The first is that many pulses with small amplitude are found on the electric field waveform. We attribute these small pulses with very short time intervals from the nearby large pulses to the leader branching process. As in frame-a and frame-d of the Fig. 3, the simultaneous development of multiple space leaders and the associated branching is clear, meanwhile the electric field in Fig. 7 shows sub-pulses very close in time with the main pulses. The step marked by the green arrow in Fig. 3(d) results in the large pulse, and the reason for why the re-activation of the space leader at the right side and the emergence of the corresponding sub-branch results in a small pulse may be that the charge involved is much smaller. Qi *et al*.^13^ once found similar small-amplitude pulses between the larger step pulses, they interpreted such small pulses as impulsive processes occurring between steps. The second fact is that multi-branches and the alternate stepping of the leader make the impulsive interval much smaller than the actual step cycle time. As revealed by the optical images, the step cycle time for an individual channel branch may reach up to 15–20 μs, while the impulsive interval in the E-field waveform can be as short as ~5 μs, even though those small pulses are not considered.

It is concluded that some space leaders fail to make connection with the original tip at first, and the drop of electric field around the original tip will lead to the interruption of these space leaders. Rationally, the electric field in the area will not significantly enhance again as the leader propagate down further. So, what factor promote the re-activation of those ever-extinguished space leaders is not very clear and it deserves further study. Meanwhile, the branching of leader occurs frequently during the stepping development, and most of the branching-attempts propagate just a quite short distance from the node and abort sooner or later. Then an important issue is that for the newly formed branches, which will survive and what factors influence the branch survival. For an objective explanation of these questions, more observations with sufficient spatial and temporal resolution are required. In addition, the laboratory studies with new observation or the re-investigation of existing data may also help to shed light on the situation.

## Method

The high-speed images were obtained in the summer season of 2013, at the SHAndong Triggering Lightning Experiment (SHATLE), by using two high-speed cameras of Phantom M310 and V711. V711 was equipped with a Nikon 14-mm lens at f/2.8, with spatial resolution of 480 × 64 pixels at operation speed of 180,000 fps (frames per second) and exposure time of 5.18 µs. M310 was configured with a Nikon 28-mm lens at f/1.8 and operated at a slower speed with a relatively lager field of view (11,000 fps, 768 × 320 pixels, and 40 µs exposure time). The manufacturer of the digital cameras, Vision Research Inc., provide the operating software to control the equipment, record the data, and display the images (http://www.phantomhighspeed.com/Service-Support/PCC-Software). The images of lightning flashes in Figs 1, 2 and 3 are generated by displaying the observation results with the PCC software.

Figure 1a shows the sketch of the observation site with the camera location marked in red circle. Figure 1b,c give complete views (for Flash #1) of cameras M310 and V711, respectively. The red dotted box marks the overlapped region in the field of view of cameras V711 and M310 (due to a 3 m horizontal distance between the cameras, there is a very tiny difference in their angles of vision). The black dashed lines in Fig. 1a show the field angle of V711 camera. That is, the luminous events occurring within this field angle would be captured, as shown in the full view in Fig. 1c or within the red dotted box in Fig. 1b. The two flashes were located by the lightning location network that was set up and operated by the State Grid Corporation of China, giving horizontal distances of 410 m and 1030 m from the camera, respectively, as marked in Fig. 1a. The azimuth of the located stroke (from the camera) are in good agreement with the optical observation. Both flashes were partly captured within the field of vision of V711, with the grounding points terminated at left outside and right outside the field of view, respectively, as shown in Fig. 1c,e. This is consistently shown in Fig. 1a by means of the lightning location network. As a matter of fact, the black line in Fig. 1a marks the azimuth direction of Flash #1 as determined by the M310 observation (see Fig. 1b), it intersects with the blue circle of 100 m radius, indicating that the locating error for Flash #1 should be less than 100 m. The locating accuracy for this two flashes is reliable, and the horizontal distance of 410 m and 1030 m were used in the image calibration, based on which a single pixel of the image observed by V711 represents the 2-D space lengths of 0.57 m (for Flash #1) and 1.44 m (for Flash #2), respectively.
